# Photo-Crosslinked Polyurethane—Containing Gel Polymer Electrolytes via Free-Radical Polymerization Method

**DOI:** 10.3390/polym16182628

**Published:** 2024-09-18

**Authors:** Fatmanur Uyumaz, Yerkezhan Yerkinbekova, Sandugash Kalybekkyzy, Memet Vezir Kahraman

**Affiliations:** 1 Department of Chemistry, Faculty of Science, Marmara University, Istanbul 34722, Turkey; fatmanur.uyumaz@marmara.edu.tr; 2 National Laboratory Astana, Nazarbayev University, Astana 010000, Kazakhstan; yerkezhan.yerkinbekova@alumni.nu.edu.kz; 3 Department of Chemistry, School of Sciences and Humanities, Nazarbayev University, Astana 010000, Kazakhstan

**Keywords:** gel polymer electrolyte, UV-crosslinking, polyurethane acrylate, polyurethane methacrylate, free-radical polymerization, lithium-ion battery

## Abstract

Using a novel technique, crosslinked gel polymer electrolytes (GPEs) designed for lithium-ion battery applications have been created. To form the photo crosslink via free-radical polymerization, a mixture of polyurethane acrylate (PUA), polyurethane methacrylate (PUMA), vinyl phosphonic acid (VPA), and bis[2-(methacryloyloxy)ethyl] phosphate (BMEP) was exposed to ultraviolet (UV) radiation during the fabrication process. The unique crosslinked configuration of the membrane increased its stability and made it suitable for use with liquid electrolytes. The resulting GPE has a much higher ionic conductivity (1.83 × 10^−3^ S cm^−1^) than the commercially available Celgrad2500 separator. A crosslinked structure formed by the hydrophilic properties of the PUA-PUMA blend and the higher phosphate content from BMEP reduced the leakage of the electrolyte solution while at the same time providing a greater capacity for liquid retention, significantly improving the mechanical and thermal stability of the membrane. GPP2 shows electrochemical stability up to 3.78 V. The coin cell that was assembled with a LiFePO_4_ cathode had remarkable cycling characteristics and generated a high reversible capacity of 149 mA h g^−1^ at 0.1 C. It also managed to maintain a consistent Coulombic efficiency of almost 100%. Furthermore, 91.5% of the original discharge capacity was maintained. However, the improved ionic conductivity, superior electrochemical performance, and high safety of GPEs hold great promise for the development of flexible energy storage systems in the future.

## 1. Introduction

Lithium-ion batteries (LIBs) are a leading player in the energy storage industry, especially for portable devices. They are widely used in large-scale electrochemical energy storage applications, which are essential for the development of electric vehicle systems. This rise in use can be attributed primarily to the advantageous range of properties they provide, such as strong energy density, long operating lives, high operating voltages, low self-discharge rates, low weight, and environmental friendliness [1,2,3,4]. Although LIBs are widely recognized as highly efficient electrochemical devices with remarkable energy density, there are certain safety concerns associated with them. The primary reason for these concerns is that the organic electrolytes that are typically used in conventional lithium-ion batteries are flammable [5,6,7,8]. The use of polymer electrolytes as the electrolyte and separator in LIBs has been accepted as an achievable strategy to reduce these safety concerns [9,10].

For several decades, liquid electrolytes have held a pivotal role in electrochemical energy storage, primarily attributed to their high ionic conductivities ranging from 10^−3^ to 10^−2^ S cm^−1^ and their effective interface interactions with electrodes. However, using liquid electrolytes has brought with it some inherent risks, such as the possibility of organic electrolyte components burning and leaking. Lithium dendrite growth in the liquid medium is another notable disadvantage of liquid electrolytes used in lithium batteries. This phenomenon arises from inconsistent current distribution and is more pronounced in the case of porous separators and lithium metal electrodes. However, since solid polymer electrolytes do not depend on liquid solvents, they offer a strong way to allay these safety worries and stop the development of lithium dendrites. It is crucial to recognize that most solid polymer electrolytes that have been reported have shown considerably reduced ionic conductivities, usually between 10^−8^ and 10^−5^ S cm^−1^, and have demonstrated inadequate interactions with electrodes, which has led to a compromised cycle performance [11,12,13,14]. Their advancements have also been hampered by poor mechanical quality. In light of this, gel polymer electrolytes (GPEs), which combine the benefits of liquid and solid electrolytes, are garnering more interest due to their dual functionality as separators and electrolytes [15,16].

Various polymer matrices, including poly (ethylene oxide) (PEO), poly (vinyl alcohol) (PVA), poly (methyl methacrylate) (PMMA), polyacrylonitrile (PAN), poly (vinylidene fluoride) (PVDF), and poly (vinyl chloride) (PVC) have been studied as GPEs [16,17,18,19]. As a common ultraviolet (UV)-curable oligomer material, polyurethane acrylate possesses double bonds with high reactivity at both ends. Depending on the kind and composition of the polyol and diisocyanate employed, different structures and properties can be obtained [20,21]. The remarkable physical qualities of polyurethane acrylate (PUA) and polyurethane methacrylate (PUMA), including its fast curing time, toughness, and controllable hardness, have made it a popular choice for photocuring applications [22]. Because of its flexibility and high-density network structure, UV-curable PUA resin has been employed in electrochemical devices including lithium-ion batteries [23,24]. PUA can improve durability by increasing the interfacial binding strength of the electrolyte [25,26,27]. Due to its many benefits, including low energy consumption, high speed, high chemical stability, room-temperature operation, cheap processing costs, and environmental friendliness, UV-curing technology has been widely used. Epoxy, polyurethane, epoxy acrylate, and polyurethane acrylate derivatives have been widely used in UV-curing technologies for a variety of purposes, including advanced composite materials, surface coatings, and structural adhesives [28,29,30].

There have only been a few studies conducted on polymer electrolytes that use polyurethane as a substrate for LIBs. Among these, a notable investigation by Jiang et al. focused on GPE filled with TiO_2_ nanoparticles with good rate capability and mechanical properties. Wen et al. also conducted research on the polyurethane-based electrolytes. However, the ionic conductivity of these electrolytes was found to be lower, with values ranging from 0.3–1.59 × 10^−3^ S cm^−1^ [31,32]. Despite these drawbacks, researchers propose that the blends of polyurethane acrylate and polyurethane methacrylate improve the mechanical and electrochemical properties of gel polymer electrolytes. This proposition involves the refinement of the ultraviolet (UV)-curing technique and the incorporation of specialized polymers and adjuncts, including inorganic and hybrid compounds. This new approach has a great deal of potential to overcome current obstacles and open up new opportunities for progress [33].

This study introduces a novel category of crosslinked gel polymer electrolyte membranes. These membranes are fabricated from a composition comprising polyurethane acrylate, polyurethane methacrylate, vinyl phosphonic acid (VPA), and bis[2-(methacryloyloxy)ethyl] phosphate (BMEP). Upon activation with liquid electrolytes, these membranes can serve as gel polymer electrolytes within the context of LIBs. The integration of PUA-PUMA and the phosphate-incorporating constituents is achieved through free-radical polymerization. Free-radical polymerization initiates polymerization by generating free radicals that react with monomers to form polymer chains. These chains grow with the addition of more monomers, resulting in a polymer network [34]. The phenomenon of free-radical polymerization, occurring between the acrylate groups and phosphate groups present in VPA and BMEP, engenders the creation of a crosslinked polymeric network. A number of favorable characteristics are displayed by the resulting polymeric network, such as a high crosslinking density and increased resistance to chemical influences. All of these characteristics make it feasible for a variety of uses, most notably in the field of LIBs. The crosslinking process assisted by UV irradiation is remarkable as it gives the membrane a noticeable increase in mechanical strength and flexibility, especially after impregnation with liquid electrolytes. Among the gel polymer electrolytes examined, compositions containing 10% BMEP content are particularly noteworthy. This specific formulation distinctly demonstrates the highest measured ionic conductivity, quantified at a level of 1.83 × 10^−3^ S cm^−1^. This value appears to be significantly greater than the ionic conductivity measurement of the commercially available Celgard2500 separator, which is 0.36 × 10^−3^ S cm^−1^ [35]. The suggested GPE system offers superior lithium-ion conductivity compared to commercial separators, making it a promising choice for flexible LIBs.

## 2. Materials and Methods

### 2.1. Materials

Aliphatic polyurethane acrylate resin (SU 574B) was supplied by Soltech (Seoul, Republic of Korea); Aliphatic polyurethane methacrylate resin (UM-2375C/H) was supplied by MCT Chem (Istanbul, Türkiye); and vinyl phosphonic acid, bis[2-(methacryloyloxy)ethyl]phosphate, and 2-hydroxy-2-methylpropiophenone (Darocur^®^ 1173) were supplied by Sigma Aldrich (Amsterdam, The Netherlands). The materials employed for conducting the lithium-ion battery (LIB) experiments, namely, lithium metal foil with a purity exceeding 99.9%, Lithium hexafluorophosphate (LiPF_6_) solution in a solvent mixture of Ethylene carbonate/Dimethyl Carbonate/Diethyl Carbonate (EC/DMC/DEC) in a ratio of 1:1:1 (*v*/*v*/*v*) at a concentration of 1.0 M, designated as battery grade, were also sourced from Sigma-Aldrich, Netherlands. Furthermore, the commercial membrane known as Celgard 2500 was acquired from Celgard LLC (Charlotte, NC, USA).

### 2.2. Preparation of Gel Electrolyte

By combining a UV crosslinking method with free-radical polymerization, polyurethane acrylate, polyurethane methacrylate, vinyl phosphonic acid, and bis[2-(methacryloyloxy)ethyl)] phosphate were used as ingredients to create gel polymer electrolytes. In order to ensure complete homogenization of PUA, PUMA, VPA, and BMEP in various stoichiometric mass ratios, the process first required stirring at room temperature. Subsequently, 2-hydroxy-2-methylpropiophenone (HMPP) was employed as a photoinitiator, constituting 3% by weight of the solution. The resulting mixture was then cast onto a Teflon plate using a doctor’s blade to form polymer membranes. To initiate crosslinking, a UV photoreactor (Luzchem, Ottawa ON, Canada) with an LED lamp emitting light at a wavelength of 365 nm was used to cure the gel on Teflon plate with UV radiation for 3 min. Subsequent to the UV exposure, the crosslinked polymer membranes were readily detached from the Teflon plate and underwent a 12 h drying process at 40 °C within a vacuum oven. The GPEs were subsequently prepared by immersing these membranes in a liquid electrolyte, specifically a 1 M LiPF_6_ solution in EC/DMC/DEC with a volumetric ratio of 1:1:1 (*v*/*v*/*v*), for a period of 24 h within a glove box. Following this immersion, the membranes were punched into small discs measuring 19 mm in diameter. The GPEs were made suitable for the examination of their electrochemical characteristics by carefully removing any extra liquid electrolyte with filter paper.

### 2.3. Characterization

#### 2.3.1. Fourier Transform Infrared Spectra

Fourier transform infrared spectroscopy (FTIR) with Perkin Elmer Spectrum100 (Shelton, CT, USA) was used to study the chemical composition and structure of the materials. The measurement was performed with the ATR-FTIR (Attenuated total reflectance-FTIR) technique, covering a wide wave number range of 4000–400 cm^−1^.

#### 2.3.2. SEM Analysis

Germany’s EDX ZEISS Crossbeam 540 scanning electron microscope (SEM) was used to examine the membranes’ morphology and surface microstructure. An automatic sputter coater Quorum Q150T (Lewes, UK) was used to coat the SEM samples with gold in order to reduce the effects of charging.

#### 2.3.3. Thermogravimetric Analysis

Thermogravimetric analysis (TGA), on Perkin Elmer STA 6000 (Shelton, CT, USA) equipment was used to analyze the thermal stability of the crosslinked membranes. The measurements, which included a temperature range of 30 to 600 °C with a heating rate of 10 °C min^−1^, were performed in a dry nitrogen atmosphere.

#### 2.3.4. DSC Analysis

Using a Perkin Elmer Diamond DSC instrument, differential scanning calorimetry (DSC) measurements were made on the polymer membranes. The nitrogen environment used for the DSC analysis has a 10 °C min^−1^ heating rate.

#### 2.3.5. Electrolyte Uptake of Membranes

Electrolyte uptake of membranes was analyzed using a commercially available LiPF_6_ electrolyte solution dissolved in a mixture of EC/DMC/DEC (1:1:1 *v*:*v*:*v*). The membranes were prepared by submerging them in a LiPF_6_ solution for two hours at room temperature in a glovebox filled with argon. The membranes were then dried to get rid of any extra solution. Before and after submersion in the electrolyte solution, the membranes’ weights were measured, and the amount of electrolyte absorption was estimated using the supplied formula.

$$A\left(\%\right)=\frac{w_{2}-w\;}{w_{1}}\times 100\%$$

The membrane weight, defined as *w*_1_ and *w*_2_, was measured before and after the immersion procedure.

#### 2.3.6. Electrolyte Absorption

The relative absorption ratio (*RA*) was calculated in order to assess the electrolyte’s long-term retention in the membrane. After four hours, the membrane was submerged in the electrolyte solution until it reached saturation. After that, a standard load was applied to the swollen membrane, and weight changes were monitored for a predetermined amount of time. A mathematical expression that was provided was used to calculate the *RA*:

$$RA=\frac{w_{m}}{w_{m,\;s}}$$

The formula is related to two different variables: *w_m_*_,*s*_, which is the mass of the membrane at the moment when the electrolyte has fully saturated the membrane; and *w_m_*, which is the mass of the membrane after the 5N load has compressed the saturated membrane for a predefined period of time.

#### 2.3.7. Gel Fraction

The gel fraction values were examined in order to evaluate PP membranes’ chemical stability. Briefly, 20 mL Dimethyl carbonate (DMC), an organic solvent, was used for this experiment. Samples, which were 19 mm in diameter, were left to soak for 24 h at room temperature. Following the removal of any remaining solvent, the sample was given a 24 h drying period in a vacuum oven set at 60 °C. The resulting gel fraction (*GF*) was then computed.

$$GF\;\left(\%\right)=\frac{w_{remained}}{w_{total}}\times 100\%$$

Prior to and following the test, the sample’s weight was determined; the results are given as *w_total_* and *w_remained_*, respectively.

#### 2.3.8. Porosity

The porosity of the membranes was determined by an absorption method using a solution of n-butanol. The weight of the crosslinked membrane was measured both before and after it was immersed in n-butanol for two hours. Taking into account the mass of the membrane, the porosity (*P*) was calculated using the given formula after carefully wiping off the excess n-butanol with a filter paper.

$$P\;\left(\%\right)=\frac{w_{a}-w_{p}}{\;\rho bV}\times 100$$

where *w_p_* and *w_a_* stand for the membrane’s masses prior to and after immersion, *ρb* for the density of n-butanol, and *V* for the membrane’s geometric volume.

#### 2.3.9. Tensile Stress–Strain Test

Tensile stress–strain tests were regularly utilized to evaluate the mechanical properties of the membranes. The studies were conducted using material testing equipment ZwickRoell Z010/TN2S (Ulm, Germany) with a displacement rate of 10 mm/min. The samples were prepared by cutting the polymer membranes into 1 cm wide by 5 cm long strips. All of the samples’ membrane thicknesses were found to be 25 μm using a caliper. The microtensile tester was then connected to the created membranes in order to perform the tests.

#### 2.3.10. Crosslinking Density

The crosslinking density of the membranes was calculated according to the Elastic Modulus method using the stress–strain test.

$$cd=\frac{E}{3RT}$$

where *cd* is the crosslinking density, *E* is the elastic modulus of the polymer, *R* is the universal gas constant (8.314 J/molK), and *T* is the absolute temperature at which the modulus was measured (in Kelvin).

### 2.4. Electrochemical Characterization

#### 2.4.1. Electrochemical Impedance Spectroscopy (EIS)

Electrochemical impedance spectroscopy (EIS) was used to assess the ionic conductivities of membranes introduced with a commercial liquid electrolyte. These experiments were conducted utilizing an alternating current (AC) amplitude of 5 mV, encompassing a frequency range from 0.1 Hz to 1 MHz. Within a coin cell configuration, a gel polymer electrolyte was interposed between two blocking electrodes, composed of stainless steel (SS). Subsequently, the determination of ionic conductivity was executed by employing the provided mathematical formulation.

$$\sigma =\frac{h}{RbS}$$

In the presented mathematical equation, the symbol *σ* denotes the ionic conductivity, *Rb* is the bulk resistivity, *h* is the membrane thickness, and *S* is the contact area between the membrane and the SS electrodes.

#### 2.4.2. Linear Sweep Voltammetry

The electrochemical stability evaluation of the PP membranes was carried out by means of linear sweep voltammetry (LSV) experiments performed in a coin cell configuration of type CR2032. Li metal was used as the reference electrode, while SS was used as the working electrode. The potential voltage range at room temperature was set from 2.0 to 6.0 V with a scan rate of 0.1 mV s^−1^. LSV and EIS data were evaluated using a Bio-Logic Instruments, VMP-3 potentiostat/galvanostat (Isère, France).

#### 2.4.3. Electrochemical Performance Tests

The electrochemical performance was investigated by constructing CR2032 coin cells using Li anode, LiFePO_4_ cathode, and commercial LiPF_6_ electrolyte. Using a battery testing instrument Optosense OPT8-MA (Winter Park, FL, USA), the cycling and C-rate performance was assessed by varying the C-rate within a voltage range of 2.7–4.2 V.

## 3. Results

**Figure 1:**
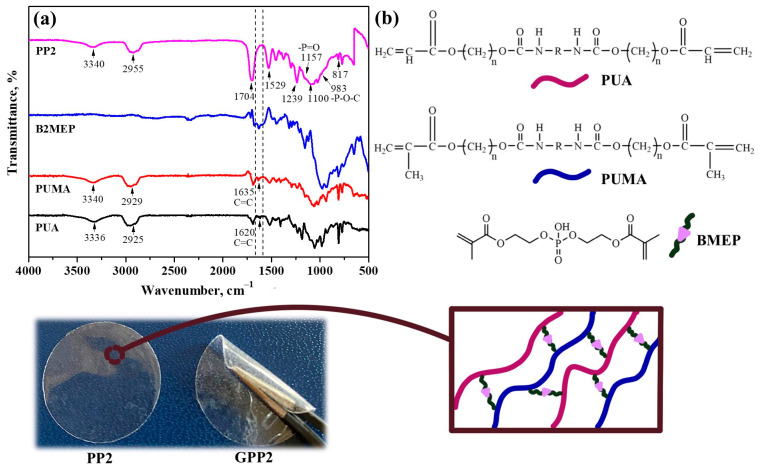
(**a**) The PP2 membrane’s FTIR spectrum and (**b**) the PP2 membrane’s composition and photo illustration.

Gel electrolyte membranes were fabricated from the solution of PUA, PUMA, vinyl phosphonic acid, and Bis [2-(methacryloyloxy)ethyl)] phosphate by the UV-curing technique. PUA-PUMA-based crosslinked membranes were created after crosslinking occurred by free-radical polymerization through the addition of a phosphate group (P=O)-containing compound to the structure and their chemical structure was investigated by FTIR spectroscopy (Figure 1). This reaction eliminates the need for the use of hazardous chemicals and can be initiated by various methods such as high temperature or UV-light exposure. It has significant value as it proceeds very quickly under moderate reaction conditions. The assembly of free radicals using radical initiators and subsequent modification of these radicals with monomer groups are the fundamentals of this type of polymerization. Adjustments were made to the ratio of substrates in the PP membranes in order to improve the membrane’s physical and electrochemical characteristics (Table 1). This change ensured great stability in organic liquid electrolyte solutions.

**Figure 2:**
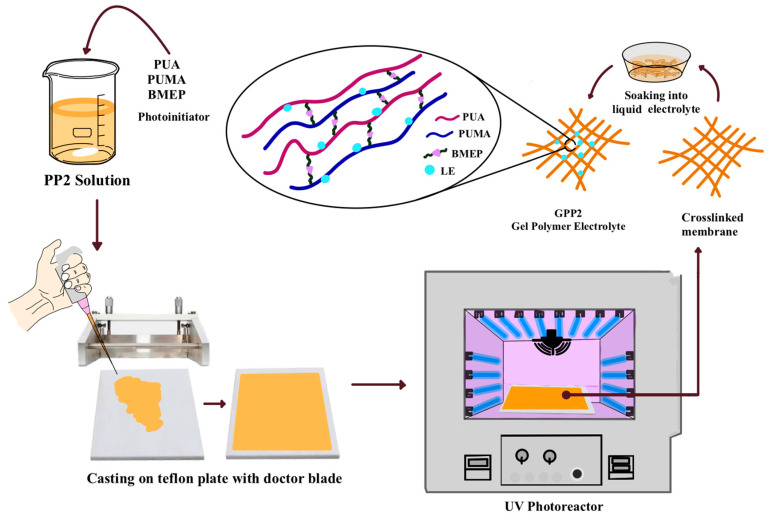
Schematic representation of the synthesis of the UV-cured crosslinked membrane PUA/PUMA/BMEP (GPP2) gel polymer electrolyte.

Overall, the FTIR peaks obtained from the membrane can serve as valuable indicators of the molecular composition and bonding characteristics resulting from the UV-induced polymerization process. The following FTIR spectra are shown in Figure 1a: the N-H stretch of PUA and PUMA at 3340 cm^−1^; C-H stretching vibrations at 2955 cm^−1^; the C=O stretching peak caused by ester bond at 1704 cm^−1^; the -NH deformation peak at 1529 cm^−1^; at 1239 cm^−1^, the peak of C-O-C stretching; at 1157 cm^−1^, the stretching peak of BMEP’s P=O phosphate group [36]; at 1100 cm^−1^, the C-N peak; at 983 cm^−1^, the stretch of the P-O-C bond peak; and the C-H peak is located at 817 cm^−1^. The disappearance of the C=C peak in the PP2 membrane’s spectrum proves the formation of free-radical polymerization and the presence of a crosslinked structure [37]. Synthesis of the UV-cured crosslinked membrane PUA/PUMA/BMEP (GPP2) gel polymer electrolyte scheme is given in Figure 2.

**Figure 3:**
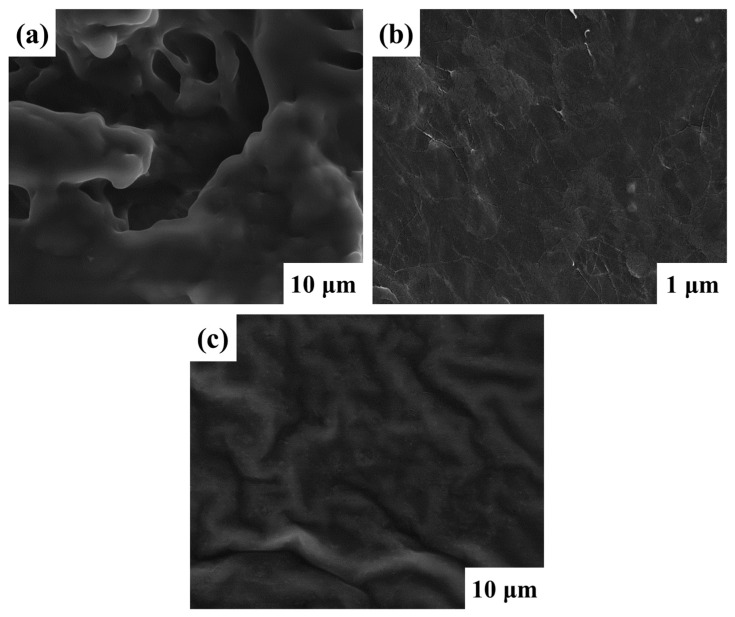
SEM images of the membranes: (**a**) PP1; (**b**) PP2; (**c**) PP3.

The Scanning Electron Microscopy (SEM) images in Figure 3, illustrate the morphological characteristics of the polymer membranes denoted as PP1, PP2, and PP3. These polymer membranes, which contain components of both BMEP and VPA, exhibit different surface porosities. Their increased ionic conductivity and increased ability to absorb electrolytes have an inherent connection to this phenomenon. The PP2 membrane’s SEM micrograph (Figure 3b), in particular, is noteworthy because it displays complex networks of interconnected molecules that resemble ropes and are connected by crosslinking interactions between multiple molecular entities. This microstructural observation serves to underscore the amorphous nature inherent to the membrane composition [38]. Clearly, there are more spaces between molecules in the polymer chains, which results in an image that highlights the gaps between the polymer moieties. This advantageous configuration creates channels that facilitate the easy diffusion of lithium ions, resulting in electrochemical characteristics characterized by high ionic conductivity.

**Figure 4:**
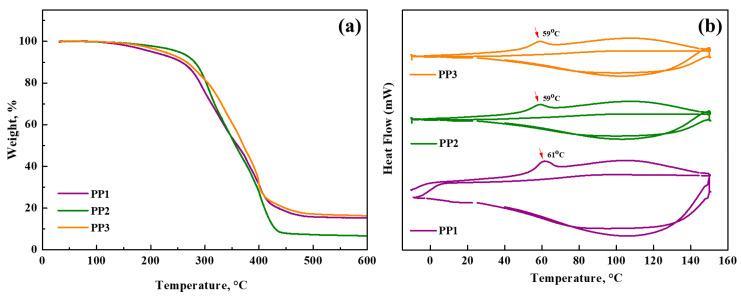
(**a**) Thermogravimetric analysis (TGA), and (**b**) Differential Scanning Calorimetry results of membranes.

Due to their remarkable thermal stability, membranes are particularly suitable for incorporation into LIBs as they contribute to the overall safety assurance of the battery. To evaluate the thermal stability of the Gel Polymer Electrolytes, Thermogravimetric Analysis (TGA) was performed under a nitrogen atmosphere using a heating rate of 10 °C min^−1^ over a temperature range of 30–600 °C. Examination of the TGA curves for the PP membranes, as shown in Figure 4a, led to the determination that the onset of degradation occurred at approximately 270 °C. The apparent weight loss ranging from 4% to 5% for all samples below 200 °C is attributed to the loss of volatile components incorporated in the polymer matrix. In contrast, PP2 and PP3 membranes containing BMEP exhibit significantly higher decomposition temperatures compared to the PP1 membrane without BMEP. This difference and increased thermal stability are attributed to the highly crosslinked structure provided by BMEP. The evaporation of both bound and unbound water molecules present in the structure is the reason behind the weight reduction in membranes, which approaches 15–18% in the 200–300 °C temperature range. All these results support the excellent thermal stability of polymer membranes, with significant thermal degradation occurring mainly above 300 °C. The safety and applicability credentials of these membranes are thus strengthened and they should be considered as reliable and suitable candidates for integration into lithium-ion batteries.

Differential Scanning Calorimetry (DSC) was employed to glass transition temperatures, utilizing a heating rate of 10 °C min^−1^ under a nitrogen (N_2_) atmosphere. The DSC investigations were conducted over two thermal cycles within hermetically sealed aluminum pans, encompassing a temperature range from −10 °C to 150 °C. The glass transition temperature (Tg) was deduced as the midpoint of the gradual transition occurring during the second heating cycle (Figure 4b). In this context, the expected effect of a substantial crosslinked network can lead to high Tg values [39]. The ionic conductivity of gel polymer electrolytes is significantly affected by the glass transition temperature. The claim that polymer electrolytes with certain chemical compositions can achieve high ionic conductivity independent of Tg values is supported by various studies [40]. Consequently, the determined Tg values for the PP1, PP2, and PP3 membranes were observed to be 61 °C, 59 °C, and 59 °C, respectively.

**Figure 5:**
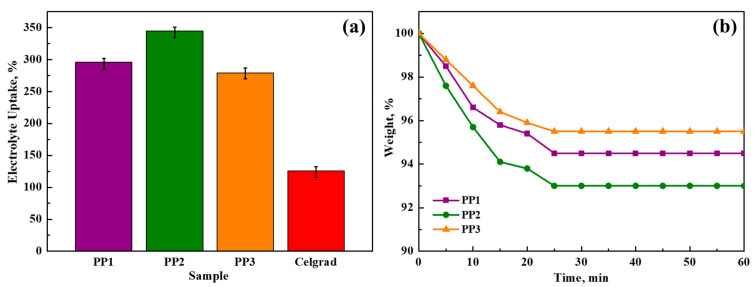
The following images show (**a**) liquid electrolyte uptake charts and (**b**) liquid electrolyte leakage test results.

The mechanism of the membrane’s liquid electrolyte uptake is illustrated graphically in Figure 5a. The lowest absorption values, 279% and 296% for PP1 and PP3 membranes, respectively, demonstrate the way these polymer membranes can absorb electrolytes better than some conventional matrices [41]. An important finding is that the PP2 membrane has the highest electrolyte uptake, with a maximum value of 345%. The membrane’s substantial porosity, which promotes effective ion transport and is crucial in increasing the ionic conductivity of gel polymer electrolytes, is responsible for the remarkable electrolyte absorption ability [42]. A battery separator with exceptional dimensional stability can enhance safety and have a positive effect on battery performance by preventing electrode contact [43]. As demonstrated by Figure 1, the PP membranes exhibited good dimensional stability following swelling with liquid electrolyte; no discernible dimensional change, asymmetry, or wrinkles were present.

Figure 5b, which depicts the findings of the electrolyte leakage studies, demonstrates that all membranes attain leakage equilibrium within the first 25 min period and that there is no further leaking after that. The hydrophilic properties and crosslinked structure of the PUA-PUMA are essential for successfully reducing electrolyte solution leakage. The crosslinked polymer membranes have a positive association with the electrolyte by successfully retaining a substantial volume of liquid electrolyte inside their structure. Due to PUA-PUMA’s strong affinity for the electrolyte solution, the polymer membranes have an amazing capacity to hold liquid. The membrane with the highest electrolyte uptake rating, PP2, has a maximum weight loss of seven weight percent.

Gel fraction measurements were used to evaluate the chemical resistance of the polymer membranes in liquid electrolyte media. For this evaluation, the membranes were immersed in dimethyl carbonate (DMC), an organic solvent contained in the liquid electrolyte, for 24 h and then dried. The resulting gel fractions were then determined using the methodologies outlined in the experimental section. Through the incorporation of vinyl phosphonic acid and bis[2-(methacryloyloxy)ethyl] phosphate into the PUA-PUMA matrix, as outlined in Table 2, a noteworthy enhancement in the chemical stability of the membranes was achieved. The chemical stabilities of the PP1, PP2, and PP3 membranes experienced substantial improvements, registering changes of 98.60%, 99.09%, and 98.18%, respectively. The crosslinking process played a pivotal role in preserving the structural integrity of the polymer matrix when exposed to organic solvents, thereby preventing its disintegration. The membranes’ notable increase in gel fraction values suggests that PUA-PUMA and VPA-BMEP have successfully crosslinked.

The introduction of pores within polymer matrices, a pivotal attribute in electrolyte systems, facilitates the permeation of liquid electrolytes through the membrane. This property increases the membrane’s ability to hold more electrolyte in addition to creating a route for ions to migrate through, which in turn raises the polymer electrolyte’s ionic conductivity with a porosity of 81.3%, PP2 demonstrated the highest porosity, as shown by the porosity values in Table 2. This increased porosity has a direct impact on the membrane’s capacity to absorb electrolytes, which is an essential property that is impacted by the material’s structural shape as well as the polymer matrix’s chemical composition. Liquid electrolytes can more easily enter the membrane structure through surface pores in the membranes, effectively stopping any leakage.

In order to demonstrate GPE’s stability and prevent lithium dendrite growth during charge and discharge cycles, the mechanical characteristics of GPE are crucial [44]. Standard tensile stress–strain test results were used to assess the mechanical properties of the crosslinked polymer membranes. The PP2 membrane had a higher Young’s modulus value than the other formulations because of the crosslinked bonds that are present in its structure and its flexibility. The Young’s modulus values for PP1, PP2, and PP3 membranes were found to be 2679, 3323, and 3011 N/mm^2^, respectively. The presence of BMEP results in a crosslinked structure that improves the membrane’s strength. BMEP is coupled with PUA-PUMA between phosphate groups. The ability of the GPEs to completely bend after swelling in the LiPF_6_ electrolyte is another way that Figure 1 demonstrates the flexibility of the GPEs. Since all evaluated samples show exceptional mechanical stability, the membranes can maintain their integrity even at thin thicknesses of about 25 μm when dry and about 30 μm when in gel form. According to the mechanical testing (Elastic Modulus Method), the crosslinking densities for PP1, PP2, and PP3 membranes were found to be 360,252, 446,853, and 404,897 mol/m^3^, respectively.

**Figure 6:**
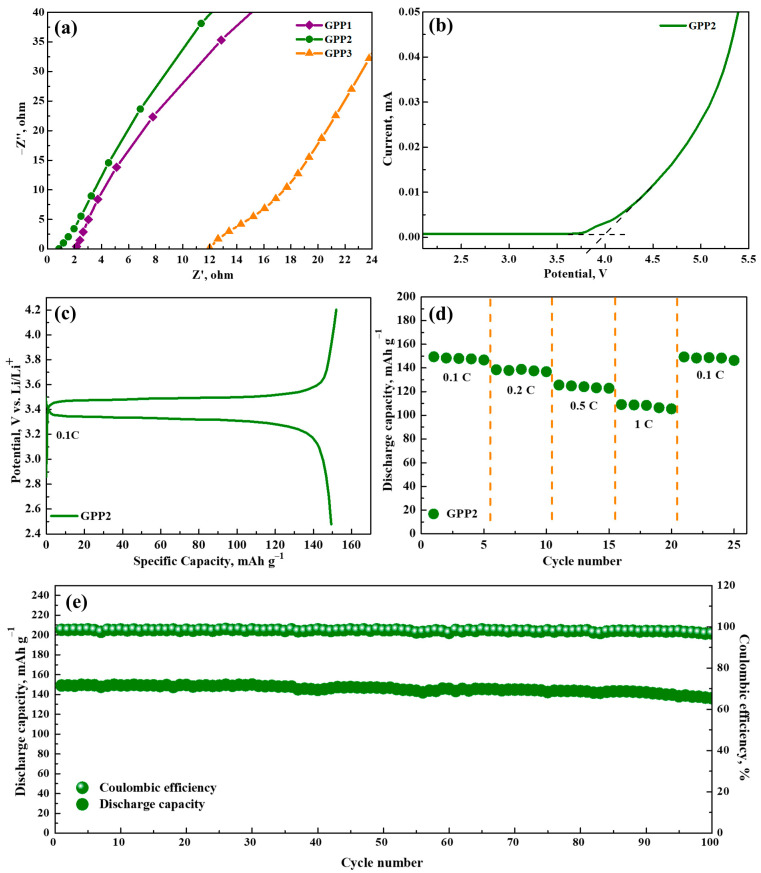
(**a**) The outcomes of electrochemical impedance spectroscopy on symmetric stainless-steel (SS) electrodes using GPP membranes. (**b**) The linear sweep voltammogram for the Li/GPP2/SS cell. (**c**) The initial charge–discharge profiles of GPP2. (**d**) GPP2 rate performance. (**e**) Galvanostatic cyclability and Coulombic efficiency of Li/GPP2/LiFePO_4_ cell.

The primary objective in the selection of electrolyte materials is ionic conductivity, a crucial parameter required for the efficient electrochemical operation of batteries. Electrochemical impedance spectroscopy experiments were conducted at ambient temperature using stainless-steel electrodes employed as blocking electrodes (SS/GPE/SS) to assess the conductive properties of gel polymer electrolytes. All of the GPE samples’ impedance spectra showed similar plots, and the bulk resistance (Rb) and the interfacial transition resistance had a significant impact on the battery’s electrochemical performance. The pronounced significance of Rb in the overall impedance values is visually depicted in Figure 6a. Using the impedance spectroscopy results, ionic conductivity values for GPEs were calculated and are listed in Table 2.

Each GPE sample exhibited an initial thickness of approximately 30 μm prior to their assembly into coin cells. The incorporation of bis[2-(methacryloyloxy)ethyl] phosphate into the GPE structure resulted in noticeable enhancements in conductivity values. This increase is explained by porosity and the increased absorption of liquid electrolytes, both of which contribute to the polymer electrolyte’s overall improvement in ionic conductivity. Notably, among the various GPE samples, the GPP2 membrane displayed the highest recorded conductivity, measuring at 1.83 × 10^−3^ S cm^−1^. This conductivity level significantly exceeds that of Celgard2500, a commercial separator membrane, which registered a conductivity of 0.36 × 10^−3^ S cm^−1^ [35]. When a higher percentage of BMEP is added to the structural framework, the crosslinking density increases significantly, which accounts for the GPP3 membrane’s reduced ionic conductivity. As a result, the absorption of electrolytes was inhibited.

Linear sweep voltammetry curves in the Li/GPE/SS blocking cell configuration at a potential range of 2 to 6 V at 25 °C are representative of the electrochemical stability window of the GPP2 electrolyte. In the scan potential range of 2 V to 3.78 V, as shown in Figure 6b, there is no observable response from the current. It is stable enough to use as the electrolyte material in lithium batteries, with an electrochemical window of 3.78 V for the GPP2 electrolyte. Our goal for the future is to fully investigate the electrochemical performance of the new polymer electrolyte, which is essential to the assembly of the entire battery cell.

Figure 6c displays the cell’s initial charge–discharge characteristics with GPP2 at a 0.1 C rate (25 °C, 2.7–4.2 V). Flat charge and discharge potential plateaus were seen in the unit cells at 3.43 and 3.33 V, respectively. At 0.1 C, the cell had initial discharge capabilities of 149 mAh g^−1^. Approximately 88% of LiFePO_4_’s theoretical capacity (170 mA h g^−1^) is represented by these values. Step-by-step variations in charging currents from 0.1 C to 1 C every five cycles were used to examine the rate capacity of the GPP2-containing cell (Figure 6d). With specific discharge capacities of 149, 138, 125, and 109 mA h g^−1^ at 0.1, 0.2, 0.5, and 1 C, respectively, the Li/GPP2/LiFePO_4_ cell is shown to have outstanding rate performance. The cell can retain its high specific capacity of approximately 149 mA h g^−1^ after undergoing five cycles at a high current density of 1 C. This is achieved when the current density decreases back to 0.1 C. Furthermore, the cell containing GPP2 maintains a 91.5% capacity retention rate and a remarkably high discharge-specific capacity of 136 mAh g^−1^ after 100 cycles (Figure 6e). The cell’s good rate performance can be attributed to high ionic conductivity, good affinity with the liquid electrolyte, and minimal formation of lithium dendrite on membrane surfaces, which results in faster lithium-ion transportation at high charge–discharge rates. A Coulombic efficiency number near 100% shows that cell processes are highly reversible and that cycle performance remains stable. The BMEP-containing electrolyte offers superior electrode interface contact and is a potential GPE system for flexible batteries, as demonstrated by all of these half-cell results.

## 4. Conclusions

In order to produce a special crosslinked gel polymer electrolyte that is advantageous for the material’s mechanical and electrochemical properties, the UV-curing method was employed. The crosslinked structure of the membranes improved their mechanical and thermal resilience while maintaining their high porosity, electrolyte absorption, and liquid-holding capacity. At 1.83 × 10^−3^ S cm^−1^, the maximum ionic conductivity of the GPP2 electrolyte was significantly higher than that of the industrial separator Celgard2500. The crosslinking of PUA-PUMA and BMEP enhanced the membrane’s mechanical strength, porosity, and ionic conductivity, while the hydrophilic characteristics of PUA-PUMA and the phosphate groups of BMEP enhanced its capacity to retain liquid. Consequently, GPP2’s electrochemical stability window is stable for up to 3.78 V. The GPP2 membrane half-cell battery showed superior electrode interface contact, a notable first discharge-specific capacity of 149 mAh g^−1^ at 0.1 C, and a capacity retention of 91.5% after 100 cycles. GPEs have a lot of potential for applications in the next generation of flexible energy storage devices because of their enhanced ionic conductivity and electrochemical performance, and exceptional thermal stability and safety.

## Figures and Tables

**Table 1:** Formulations of membranes.

Abbreviation	PP1	PP2	PP3
**GPE Membrane Formulation**	Mass (g)		
Polyurethane Acrylate	1	1	1
Polyurethane Methacrylate	4	4	4
Vinyl Phosphonic Acid	0.55	0	0
Bis[2-(methacryloyloxy)ethyl)]phosphate	0	0.55	1.25
2-hydroxy-2-methylpropiophenone	3 wt% of the total mass		

**Table 2:** Main characterizations of crosslinked membranes. * According to the manufacturer’s instructions.

Samples	Gel Fraction, %	Porosity, %	Electrolyte Uptake, %	Ionic Conductivity, S cm^−1^
PP1	98.60	78.2	296	0.70 × 10^−3^
PP2	99.09	81.3	345	1.83 × 10^−3^
PP3	98.18	76.5	279	0.12 × 10^−3^
Celgrad-2500	---	55.0 *	126	0.36 × 10^−3^

## Data Availability

All data generated or analyzed during this study are included in this published article.
